# Emerging roles of the long non-coding RNAs MALAT1 and TUG1 during differentiation of adipose tissue-derived mesenchymal stem cells towards insulin-producing cells

**DOI:** 10.1186/s13287-026-05125-y

**Published:** 2026-07-01

**Authors:** Eman F. Sanad, Alaa Ahmed Saad, Joy Rafeek, Rana Mokbel, Mayar Abdallah, Nadeen Emad, Hagar Adel Mohamed, Yasmin Alaa, Yumna Medhat Mahmoud, Dina H. Kassem

**Affiliations:** 1 Department of Biochemistry and Molecular Biology, Faculty of Pharmacy, Ain Shams University, Cairo, 11566 Egypt; 2 Drug Design Program, Faculty of Pharmacy, Ain Shams University, Cairo, 11566 Egypt

**Keywords:** Mesenchymal stem cells, Diabetes mellitus, Long non-coding RNAs, Differentiation, Insulin-producing cells

## Abstract

**Background:**

Generation of insulin-producing cells (IPCs) from stem cells provides great hope for patients with diabetes mellitus (DM). Long non-coding RNAs (lncRNAs) ignited much interest regarding their role in determining the fate of stem cells. The lncRNAs MALAT1 and TUG1 have been reported to be interrelated with β-cell dysfunction and/or DM. However, their role during generation of IPCs from stem cells has not been adequately studied. Thus, the current study aimed to investigate the role of MALAT1 and TUG1 during differentiation of adipose tissue-derived mesenchymal stem cells (Ad-MSCs) towards IPCs.

**Methods:**

Ad-MSCs were isolated from rat epididymal fat pads, characterized and induced to differentiate towards IPCs. Assessment of differentiation was done by measuring expression levels of various β-cell-related markers using RT-qPCR, as well as morphological changes, and dithizone staining. Expression levels of MALAT1 and TUG1 were also measured by RT-qPCR. Several in-silico analyses were done using RNA–protein Association and Interaction Networks (RAIN) database.

**Results:**

MALAT1 and TUG1 expression levels were significantly increased during differentiation of Ad-MSCs into IPCs as compared to control uninduced cells. Furthermore, generated networks from RAIN database revealed an interplay between MALAT1 and TUG1, and between each of them with several common targets like GAS5, HOTAIR and TP53COR1.

**Conclusions:**

The current study portrays MALAT1 and TUG1 as novel interrelated molecular mediators and important regulatory nodes enhancing differentiation of Ad-MSCs towards IPCs. Their upregulation during differentiation can be interrelated with competitive endogenous RNA (ceRNA) networks, mediating various epigenetic modifications, orchestrating signaling pathways and overcoming cellular stress during reprogramming/differentiation.

**Graphical Abstract:**

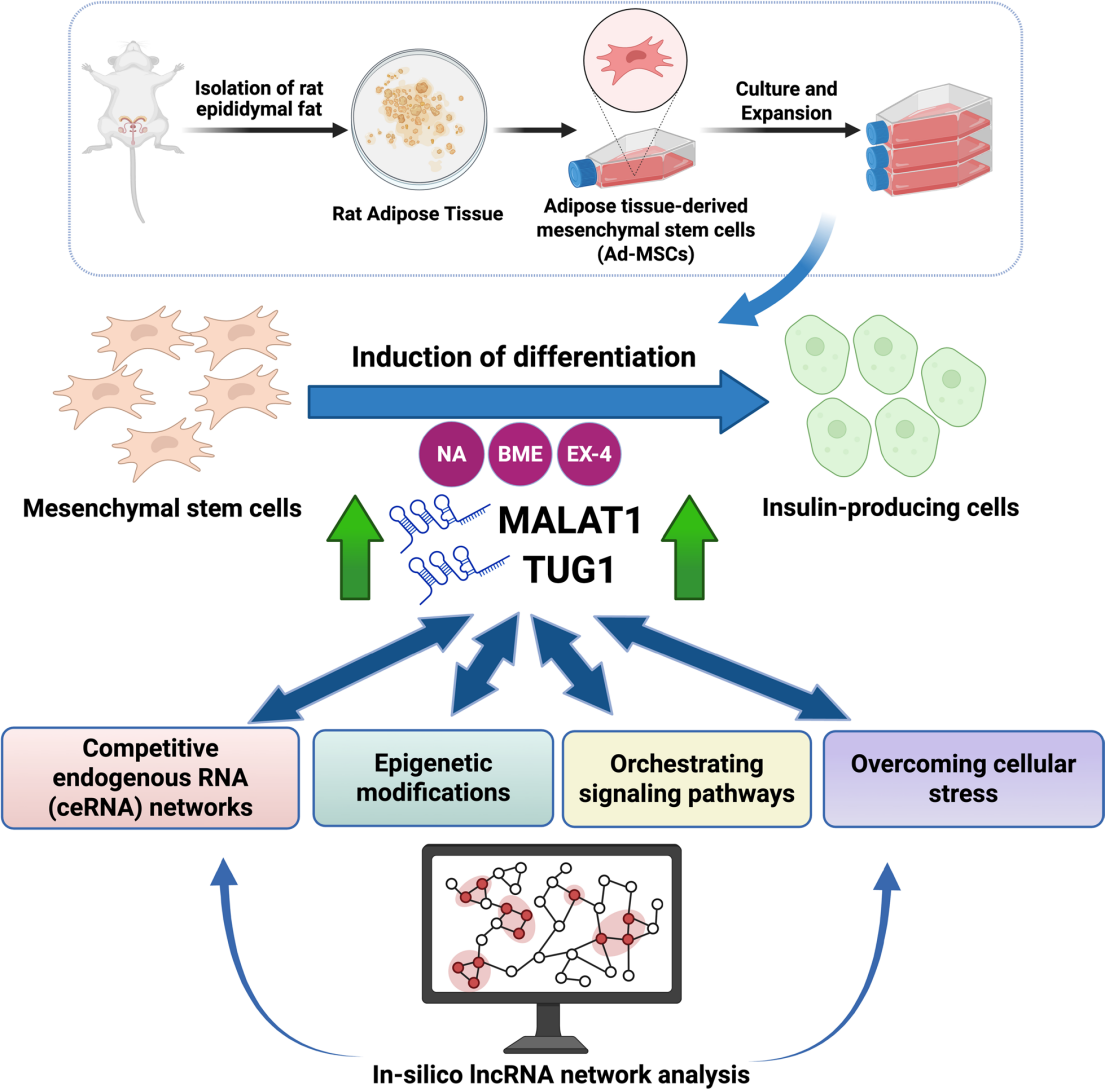

**Supplementary Information:**

The online version contains supplementary material available at 10.1186/s13287-026-05125-y.

## Background

Diabetes mellitus (DM) is a serious metabolic disorder marked by varying degrees of loss and/or dysfunction of insulin-secreting β-cells. Unfortunately, the global prevalence of DM is alarmingly rising, currently affecting approximately 589 million individuals worldwide, with Egypt ranking among the top ten most affected countries [1]. Over the past few decades, stem cell-based therapies have emerged as a promising avenue for the treatment of numerous diseases, particularly DM [2, 3]. Among various stem cell types, mesenchymal stem cells (MSCs) specifically have attracted significant attention for their broad reparative capabilities, with more than 1,500 clinical trials investigating their therapeutic applications across diverse diseases [4]. MSCs are highly valued for their ability to mediate reparative effects in diverse forms of tissue injury [5–9], coupled with several key advantages: minimal ethical concerns compared to embryonic stem cells (ESCs), relatively easy isolation procedures, robust ex-vivo expansion, together with their immunomodulatory properties and multipotent differentiation potential [10]. However, the mechanisms underlying their therapeutic and regenerative properties remain much more complex than anticipated and far from complete elucidation [5].

A particularly exciting area of research is the generation of insulin-producing cells (IPCs) from stem cells. Over the past couple of decades, various stem cell sources—including ESCs [11, 12] and MSCs [13, 14]—have demonstrated the ability to differentiate into IPCs. Despite these advances, current differentiation induction protocols remain suboptimal due to several factors such as the complexity of the signaling pathways governing pancreatic lineage specification and the variability between different types of the used stem cells [15, 16]. This highlights the need for further investigations to enhance maturation and functionality of IPCs generated from various types of stem cells. Overcoming these barriers is essential for improving cell therapy outcomes in DM and could also reveal novel therapeutic targets to stimulate β-cell neogenesis/regeneration and combat DM [17, 18].

In fact, differentiation of stem cells into IPCs employ mandatory extensive changes in gene expression orchestrating this complicated multi-factorial process. Non-coding RNAs-mediated regulatory modulations are among the prominent epigenetic mechanisms interrelated with the differentiation of MSCs [19]. Recently, there is growing evidence that long non-coding RNAs (lncRNAs) are considered key regulatory molecular factors of stem cell maintenance and differentiation [20]. LncRNAs are group of non-coding RNAs composed of more than 200 nucleotide. They act through various mechanisms and interactions with DNA, RNA, and proteins. Thus, they can regulate gene expression at transcriptional, translational, and post-translational levels [21]. Careful consideration of lncRNAs, especially those which were reported to be highly expressed/enriched in pancreatic β-cells, during differentiation of MSCs into IPCs, can indeed open new avenues for both stem cells as well as DM researchers. Recently, lncRNAs have attracted greart interest regarding their role in determining the fate of stem cells [22]. Interestingly, two of these lncRNAs, namely metastasis‐associated lung adenocarcinoma transcript 1 (MALAT1) and taurine upregulated gene-1 (TUG1) have also been reported to be interrelated with β-cell dysfunction and/or DM [23, 24]. Moreover, exendin-4, a very commonly used extrinsic factor in differentiation protocols of stem cells into IPCs, has been reported to induce the expression of MALAT1 in β-cells [23]. It’s noteworthy here that both MALAT1 and TUG1 have been reported to mediate some of their actions via sponging miRNA(s) [25, 26]. However, the role of MALAT1 and TUG1 regarding differentiation of stem cells into IPCs has not been investigated.

Accordingly, in the light of all the previous findings, we sought to isolate adipose tissue-derived MSCs (Ad-MSCs), induce their differentiation towards IPCs, and investigate the expression levels of MALAT1 and TUG1 in the isolated un-induced Ad-MSCs and during the differentiation process. This will provide a forward leap to the understanding of the role of these novel lncRNA(s), and unveil novel therapeutic targets for DM. As far as our knowledge, this is the first study investigating MALAT1 and TUG1 during differentiation of Ad-MSCs into IPCs.

## Methods

### Isolation and culture of Ad-MSCs

This study was approved by the ethical committee of Faculty of Pharmacy, Ain Shams University (Approval No. ACUC-FP-ASU-RHDIRB2020110301-REC#204, Date July 25th 2023). The current work was approved under the name “Exploring the role of some novel long non-coding RNA(s) and novel molecular mediators during differentiation of mesenchymal stem cells into insulin-producing cells”. All experiments and procedures were performed in a consistent manner with the approved guidelines. Adult male Sprague–Dawley rats, weighing 120–150 g, were obtained from the El-Nile Company for Pharmaceutical and Chemical industries, Cairo, Egypt. The rats were maintained under controlled conditions; at a 12 h light/12 h dark cycle and constant temperature (25 ± 1 °C) with free access to standard rat chow and water. The animals were acclimitized for 1 week before the experimental/isolation procedures. We have applied all the necessary precautions to avoid any harm to the animals in line with the ARRIVE guidelines 2.0.

To proceed with the isolation procedure of Ad-MSCs, three male Sprague–Dawley rats were used per isolation. The rats were anesthetized using freshly prepared cocktail of ketamine hydrochloride (33 mg/kg) and xylazine hydrochloride (13 mg/kg) intraperitoneal injection as previously described [27, 28], then afterwards euthanised by cervical dislocation. For adipose tissue collection, epididymal fat pads were aseptically collected and processed as previously described [29, 30]. Briefly, the adipose tissue was minced into small pieces and placed in 50-mL falcon tube, then washed with sterile phosphate buffered saline (PBS) (Lonza, USA). Next, the minced tissues were mixed thoroughly with PBS for 45 s, then allowed to settle down for 4 min followed by aspirating the infranatant. This step was repeated for 4–5 times until the infranatant became clear. The adipose tissue was enzymatically digested afterwards by 0.1% collagenase type I (Gibco, Thermo Fisher, USA) in PBS, and incubated at 37 °C in a shaking water bath for 1 h or until the mixture became homogenous. Next, the digested adipose tissue was vortexed for 15 s then centrifuged at 1200 rpm for 5 min. Afterwards, the supernatant was discarded and the pellet of stromal vascular fraction (SVF) was resuspended in 1% bovine serum albumin (BSA) and centrifuged at 1200 rpm for 5 min.

Afterwards, the SVF pellet was washed with PBS, and resuspended in Dulbecco's Modified Eagle Medium: F12 (DMEM:F12; Lonza, USA) supplemented with 2 mM L-glutamine (Lonza, USA), 10% fetal bovine serum (FBS), 100 U/ml Penicillin, 100 μg/ml Streptomycin and 0.25 μg/ml Amphotericin B (Lonza, USA). The cells were cultured at 37 ºC and 5% CO_2_ in a humidified atmosphere for 24 h. Next day, non-adherent cells were discarded, and the medium was completely replaced. Afterwards, the culture medium was changed every other day and the isolated Ad-MSCs were expanded in vitro. When the cells reached about 80% confluency, they were sub-cultured using 0.05% trypsin–EDTA (Lonza, USA) and designated as passage 1 (P1). All the experiments were performed on cells between P3 and P5.

### Characterization of isolated Ad-MSCs by immunophenotyping

At the third passage, Ad-MSCs were washed twice with PBS and trypsinized with 0.05% trypsin–EDTA (Lonza, USA). Then, cells were collected for identification of surface markers by flow cytometry. Briefly, about 1 × 10^5^ cells were incubated in dark for 30 min with labelled anti-rat monoclonal antibodies including fluroisothiocyanate (FITC)-conjugated anti-rat CD90 (Stem Cell Technologies, USA), phycoerythrin (PE)-conjugated anti-rat CD14 and PE-conjugated anti-rat CD34 (Beckman Coulter, USA). Then, the cells were washed and resuspended in 500 µl of PBS. Unstained cells were used as controls for gating during analysis. Cells analysis was performed by CYTOFLEX Flow Cytometer (Beckman Coulter, FL, USA) using CytExpert Software.

### Differentiation of isolated Ad-MSCs into various mesenchymal lineages

The ability of isolated MSCs to differentiate into adipogenic, osteogenic and chondrogenic lineages was investigated using mesenchymal stem cell functional identification kit (R&D systems Inc., MN, USA) according to the manufacture’s protocol. For adipogenic differentiation, the presence of lipid vesicles in differentiated cells was examined by Oil Red-O stain (Sigma-Aldrich, USA). Regarding osteogenic differentiation, the induced cells appearance was examined using inverted microscope and the induced cells were stained by Alizarin Red-S solution (Sigma-Aldrich, USA). For chondrogenic lineage, the presence of cuboid cells was explored and synthesis of proteoglycans was investigated via staining the induced cells by Alcian blue (Sigma-Aldrich, USA).

### Differentiation of Ad-MCs into IPCs

Figure 3A outlines the steps of the protocol used to induce the differentiation of Ad-MSCs into IPCs as described previously with slight modifications [29]. Briefly, 1 × 10^6^ Ad-MSCs at P3 were seeded into 6-well plates and cultured until reaching about 80% confluency. Then afterwards, induced for 48 h with 10 mmol/L nicotinamide (Sigma-Aldrich, USA) and 1 mmol/L β-mercaptoethanol (Sigma-Aldrich, USA) in 10% FBS DMEM-F12. Then for next 24 h, cells were incubated with 10 mmol/L nicotinamide and 1 mmol/L β-mercaptoethanol in high glucose (HG)-DMEM supplemented with 2.5% FBS. Cells collected after this induction step are referred to as Step-1. The remaining cells were further induced for at least 5 days with 10 nmol/L exendin-4 (Sigma-Aldrich, USA) in HG-DMEM supplemented with 2.5% FBS, 10 mmol/L nicotinamide and 1 mmol/L β-mercaptoethanol [27, 29, 31]. Cells collected after this induction step are referred to as Step-2. Un-induced Ad-MSCs were cultured in complete DMEM-F12 media supplemented with 10% FBS and kept for the whole time of differentiation protocol at same culture conditions as control. Differentiated cells were assessed through morphological investigation, dithizone staining and gene expression levels of several β-cell markers.

### RNA extraction and reverse transcriptase quantitative polymerase chain reaction analyses (RT-qPCR)

Both control un-induced Ad-MSCs and differentiated step-1 and step-2 IPCs were collected. Total RNA was extracted by TRIzol Reagent and purified by miRNeasy Mini Kit (Qiagen, USA). Briefly, cell pellets were resuspended in 1 mL TRIzol reagent then treated with 200 μL chloroform. The upper layer containing RNA was mixed with ice-cold absolute ethanol and transferred into spin column for further purification of RNA according to the manufacture’s protocol. The isolated RNA was quantified by Denovix DS-11 Spectrophotometer (DeNovix Inc., USA). About 0.5 μg total RNA was converted into cDNA using Verso TM cDNA synthesis kit (Thermo Scientific, USA). Each RT-qPCR reaction was performed by using 4 ng cDNA and Maxima® SYBR Green Master Mix (Thermo Scientific, USA). Step-One plus real time PCR machine was used (Applied Biosystems, USA). Table 1 summarizes the sequence of PCR primers used for the genes of β-cell markers; MafA NKX6.1 and Ins2, as well as target lncRNAs; MALAT1 and TUG1. β-actin was used as housekeeping gene and relative expression levels of the target genes were calculated by comparative ΔCt method as described previously [32, 33]. The relative normalized expression levels were expressed as 2 − ^ΔCt^ after normalization to the endogenous reference gene. For the investigated lncRNAs (MALAT1 and TUG1), the relative normalized expression levels are expressed as fold changes after further normalization to their expression levels in control un-induced Ad-MSCs.

**Table 1:** Forward and reverse primer sequences used for RT-qPCR

Gene	Forward primer sequence 5’-3’	Reverse primer sequence 5’-3’
*β-actin*	TGGAGAAGATTTGGCACCAC	AACACAGCCTGGATGGCTAC
*Ins2*	TGTGGGGAGCGTGGATTCTT	GTGCCAAGGTCTGAAGGTCAC
*NKX6.1*	ACACCAGACCCACATTCTCCG	ATCTCGGCTGCGTGCTTCTT
*MafA*	CACATTCTGGAGAGCGAGAAG	TCTCGTATTTCTCCTTGTACAGGTC
*MALAT1*	AAAGCAAGGTCTCCCCACAAG	GGTCTGTGCTAGATCAAAAGGCA
*TUG1*	TGCCACCAGCACTGTCACT	ACGGTCCAGGTGAATGAACA

### Dithizone staining

Control un-induced Ad-MSCs and differentiated IPCs were assessed by Dithizone (DTZ) staining to confirm IPCs differentiation as described previously [29, 34]. DTZ stock solution was prepared by dissolving 50 mg of DTZ (Sigma Aldrich, USA) in 5 ml of dimethyl sulfoxide (DMSO) and stored in dark at – 20 °C. A DTZ working solution was prepared by diluting the stock solution 1:100 in culture medium and then filtered through a 0.2-µm nylon filter. To each well in 6-well plate, 3 mL of working solution was added and incubated for 2 h at 37 °C. After three times washing with PBS, the crimson red-stained cells were investigated under an inverted microscope.

### Analysis for lncRNAs MALAT1 and TUG1 interaction networks

Interaction networks for the lncRNAs MALAT1 and TUG1 were generated using the RAIN (RNA–protein Association and Interaction Networks) tool (v1.0) within the STRING platform. RAIN integrates known and predicted associations of RNA molecules with proteins and other RNAs by combining data from experimental sources, curated databases, text mining, and co-expression analyses. For each lncRNA of interest, we input the gene name as a query, and RAIN retrieved the top predicted and experimentally supported interactors, including other lncRNAs and protein-coding genes relevant to the target lncRNA. The resulting networks were visualized in STRING with confidence scores and edge thickness indicating the strength of associations, while color coding reflected the molecular class of interactors. Only interactions with high confidence scores (STRING confidence > 0.7) were considered. These RAIN-generated networks provided an overview of the potential regulatory landscape of MALAT1 and TUG1, facilitating the identification of shared partners and possible cooperative mechanisms in cellular processes such as transcriptional regulation, chromatin remodeling, and cell cycle control.

### Statistical analyses

Data were presented as mean ± standard error of mean. Comparisons between the means of two groups or more were performed by t-test or one-way ANOVA, respectively. All statistical analyses were carried out by the GraphPad Prism 10 (GraphPad Software, USA). A *p-*value < 0.05 was considered statistically significant.

## Results

### Isolated cells from rat epidydimal fat pads exhibit all MSCs characteristics

**Fig. 1:**
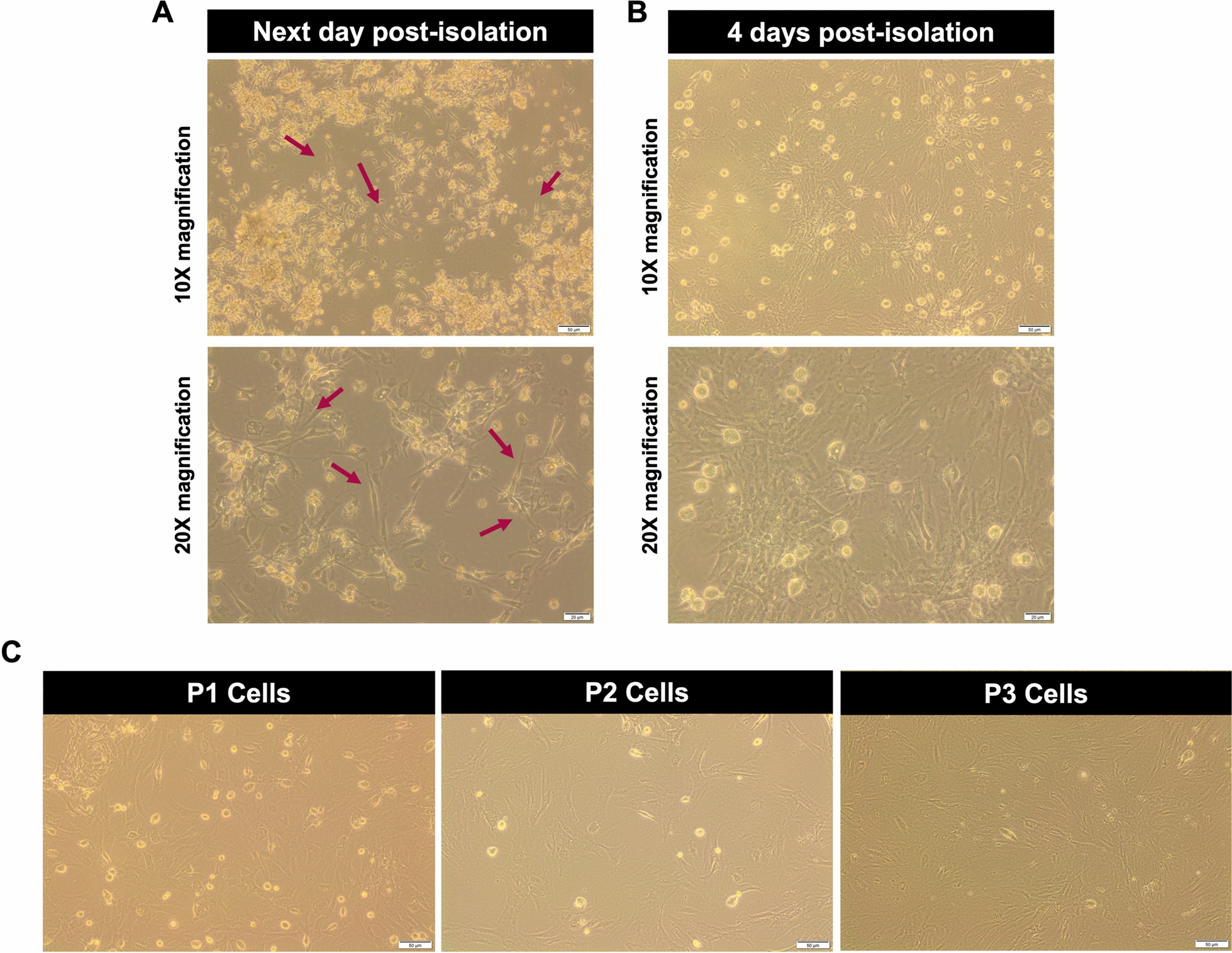
Photomicrographs showing the isolated Ad-MSCs. **A** Isolated cells exhibiting fibroblast-like, plastic-adherent morphology start to appear the next day following isolation. **B** Over time, isolated Ad-MSCs with plastic-adherent fibroblast-like morphology proliferate and increase in number, reaching a more homogenous fibroblast-like population. **C** Phase contrast images of isolated Ad-MSCs displaying homogenous population of fibroblast-like cells after first passage (P1) which continues till the third passage (P3), scale bar = 50 μm

**Fig. 2:**
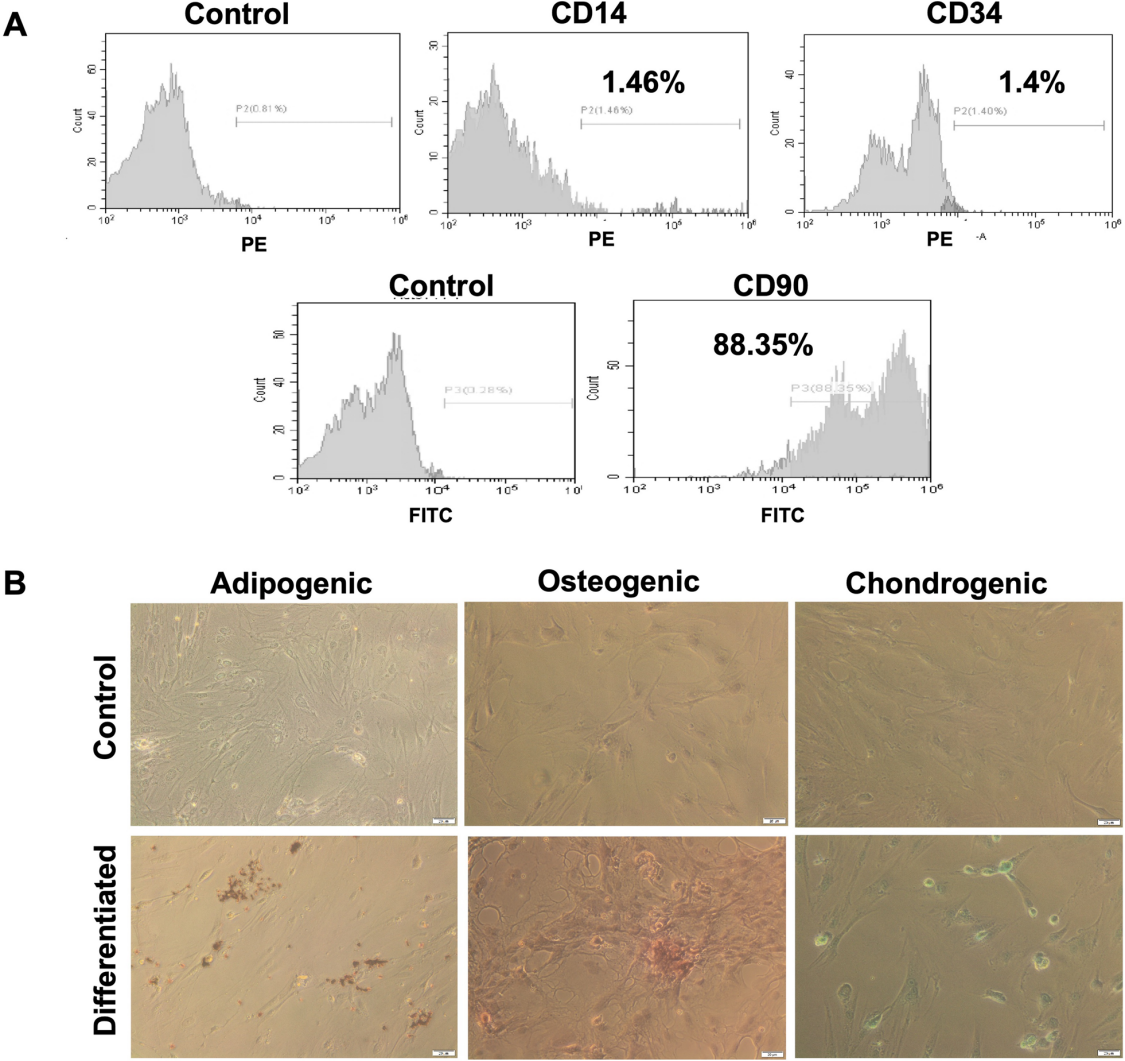
Characterization of isolated Ad-MSCs. **A** Immunophenotyping for isolated Ad-MSCs, cells were labeled with either phycoerythrin (PE)- or fluroisothiocyanate (FITC)-labelled antibodies and examined by flow cytometry. The immunophenotyping examination of Ad-MSCs showed negative/low expression of CD14 and CD34, while CD90 expression was highly positive. **B** Isolated Ad-MSCs can differentiate into the three mesenchymal lineages, namely; adipocytes (where oil droplets are stained by oil red), osteocytes (stained by Alizarin Red-S stain), and chondrocytes (stained by Alcian 8GX Blue), as compared to control uninduced cells, scale bar = 20 μm.

Isolated Ad-MSCs exhibiting plastic-adherent fibroblast-like morphology that started to appear next day to isolation and became more homogenous by time and passaging as shown in Fig. 1. According to the international society for cell and gene therapy (ISCT) [35], isolated cells displayed all characteristics of MSCs. Beside their fibroblastic plastic-adherent morphology, immunophenotyping analyses showed positive expression of CD90 (mesenchymal marker) with 88.35% and negative expression of CD45 (leukocyte specific antigen) with 3.03%, CD14 (monocytes marker) with 1.46% and CD34 (hematopoietic stem cell marker) with 1.40% as shown in Fig. 2A. Moreover, Ad-MSCs showed multipotent differentiation potential into adipogenic, osteogenic and chondrogenic lineages, as observed in Fig. 2B. These results indicate a relatively homogenous mesenchymal phenotypic population of Ad-MSCs.

### Assessment of differentiation of Ad-MSCs towards IPCs

**Fig. 3:**
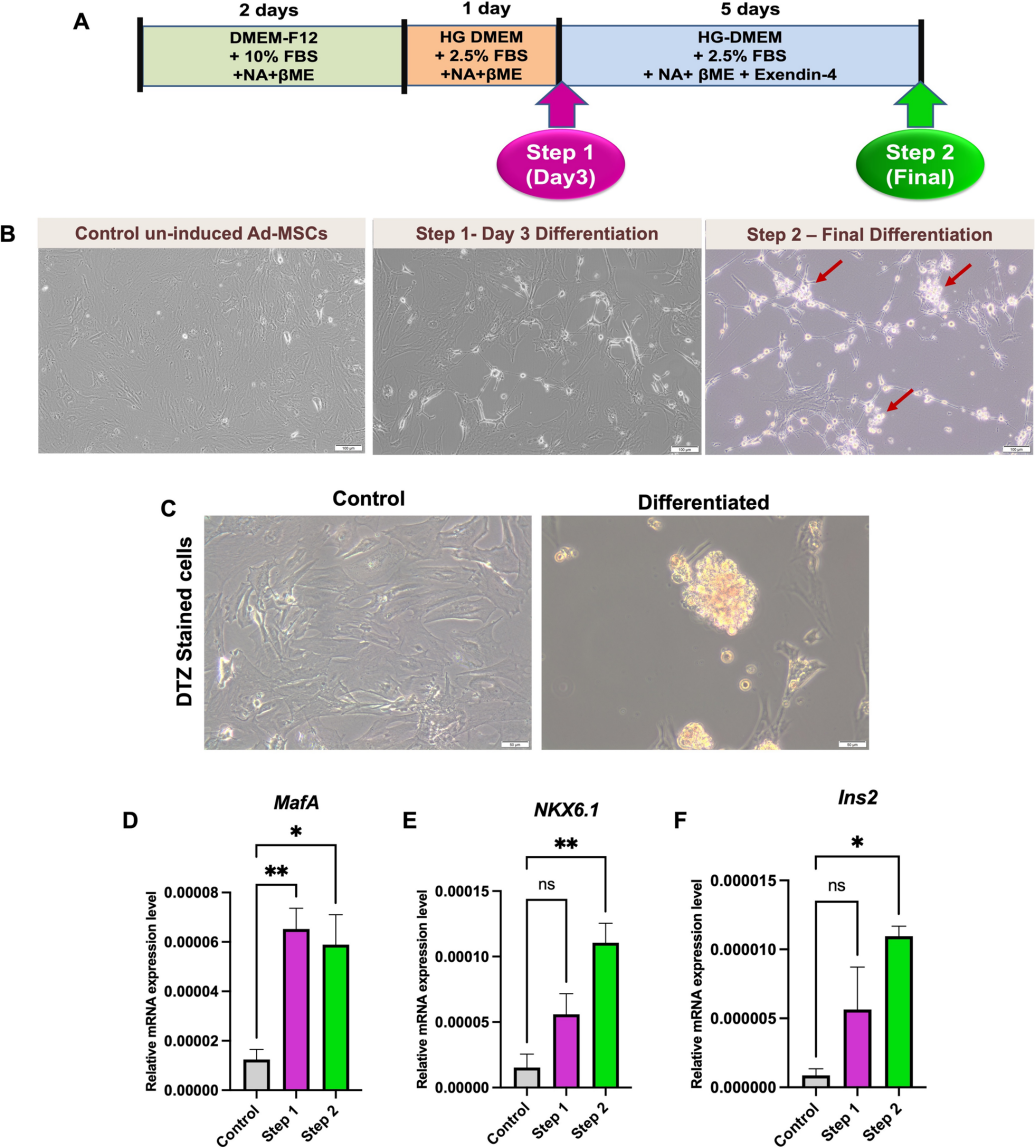
Differentiation of isolated Ad-MSCs towards IPCs. **A** Schematic presentation of the differentiation induction protocol used to generate IPCs from Ad-MSCs. **B** Photomicrographs for the induced cells at each stage during the differentiation induction of Ad-MSCs towards IPCs. Upon differentiation, cells tend to lose their fibroblastic morphology and to form aggregates/clusters. **C** Photomicrographs showing that generated IPCs exhibit positive crimson red staining by DTZ compared to control un-induced Ad-MSCs, scale bar = 50 μm. **D**–**F** Gene expression analyses by RT-qPCR for several β-cell markers; *MafA, NKX6.1,* and *Ins2* in differentiated IPCs as compared to control uninduced Ad-MSCs. Results are presented as mean ± standard error of mean (n = 3). * Means are significantly different from control uninduced Ad-MSCs at *p* < 0.05. ** Means are significantly different from control uninduced Ad-MSCs at *p* < 0.01. *NS* no significant difference from control uninduced Ad-MSCs. *β-ME* beta-mercaptoethanol, *DMEM* Dulbecco’s modified Eagle’s medium, *Ex-4* exendin-4, *FBS* fetal bovine serum, *HG* high glucose, and *NA* nicotinamide

Upon differentiation of Ad-MSCs into IPCs, the cells started to lose their fibroblastic morphology and plastic-adherence property of MSCs and tended to form aggregates which increased by the end of the induction protocol as shown in Fig. 3B. Furthermore, some cells tended to detach and started to grow in suspension, in contract to un-induced Ad-MSCs which retained fibroblast-like adherent morphology throughout the period of the induction protocol. In addition, the generated IPCs showed positive crimson red DTZ staining while un-induced control Ad-MSCs showed negative DTZ staining as illustrated in Fig. 3C.

The gene expression analyses revealed induction of several β-cell markers upon the differentiation of Ad-MSCs into IPCs as shown in Fig. 3D–F. Both *MafA* and *NKX6.1* showed significantly elevated expression levels in differentiated cells at step-1 and step-2 as compared to control uninduced cells. Also, *Ins2* expression levels started to increase at step-1, reaching significantly increased levels in differentiated IPCs at step-2 compared to control uninduced Ad-MSCs. These findings indicate the successful differentiation of Ad-MSCs towards IPCs.

### The expression levels of MALAT1 and TUG1 are significantly induced during differentiation of Ad-MSCs into IPCs

**Fig. 4:**
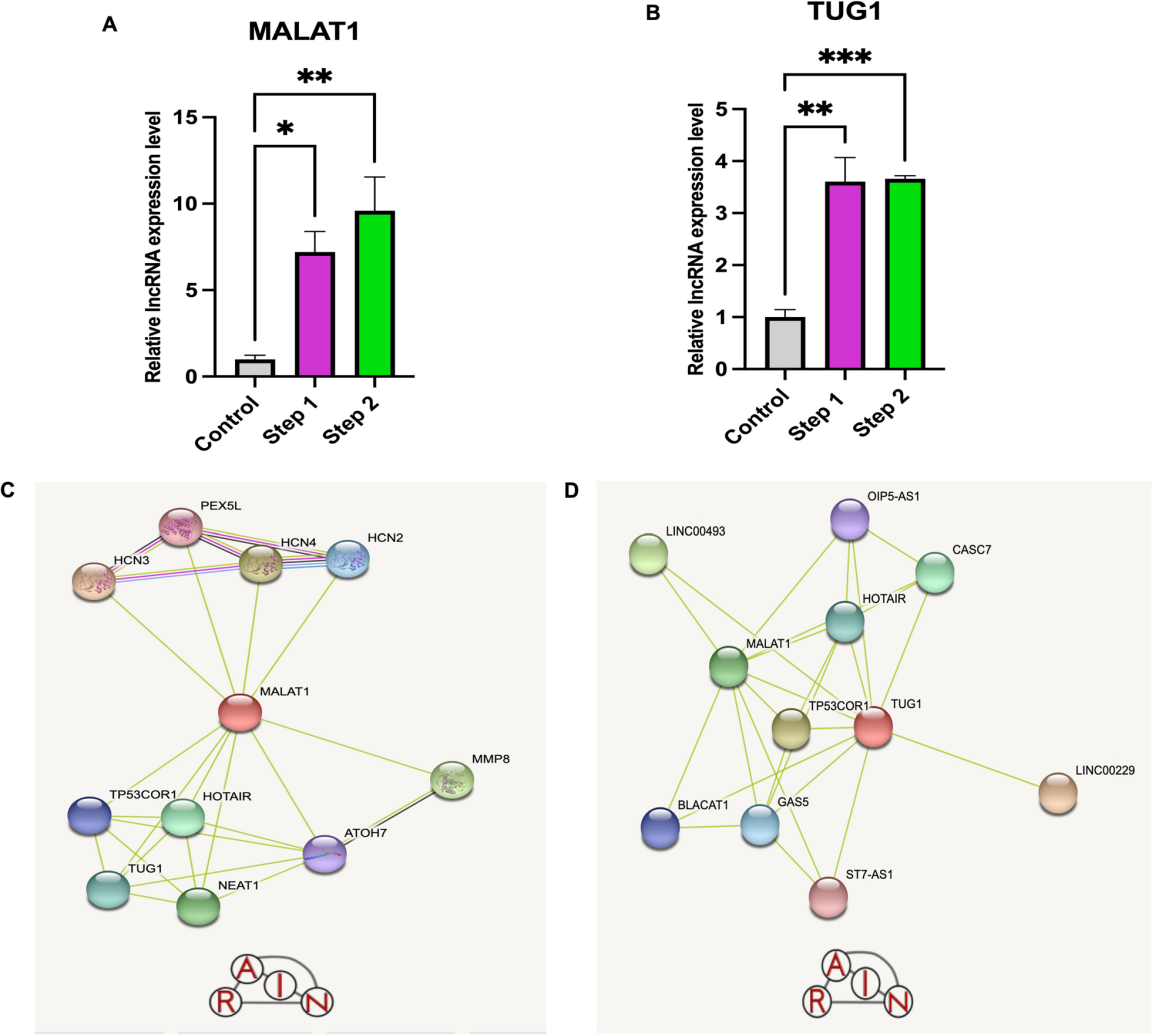
Significant elevation of expresssion levels of the lncRNAs MALAT1 and TUG1 during differentiation of Ad-MSCs towards IPCs. **A** Gene expression analysis by RT-qPCR for MALAT1 in differentiated IPCs as compared to control uninduced Ad-MSCs. **B** Gene expression analysis by RT-qPCR for TUG1 in differentiated IPCs as compared to control uninduced Ad-MSCs. Results are presented as mean ± standard error of mean (n = 3). * Means are significantly different from control uninduced Ad-MSCs at *p* < 0.05. ** Means are significantly different from control uninduced Ad-MSCs at *p* < 0.01. *** Means are significantly different from control uninduced Ad-MSCs at *p* < 0.001. **C** Interaction network for the lncRNA MALAT1 generated by RNA–protein Association and Interaction Networks (RAIN). **D** Interaction network for the lncRNA TUG1 generated by RNA–protein Association and Interaction Networks (RAIN). *ATOH7* Atonal BHLH Transcription Factor 7, *BLACAT1* Bladder Cancer-Associated Transcript 1 (Non-Protein Coding), *CASC7* Cancer Susceptibility Candidate 7, *GAS5* Growth Arrest Specific 5, *HCN2* Hyperpolarization Activated Cyclic Nucleotide Gated Potassium Channel 2, *HCN3* Hyperpolarization Activated Cyclic Nucleotide Gated Potassium Channel 3, *HCN4* Hyperpolarization Activated Cyclic Nucleotide Gated Potassium Channel 4, *HOTAIR* HOX Transcript Antisense RNA, *LINC00229* Long Intergenic Non-Protein Coding RNA 229, *LINC00493* Long Intergenic Non-Protein Coding RNA 493, *MALAT1* Metastasis-Associated Lung Adenocarcinoma Transcript 1, *MMP8* Matrix Metallopeptidase 8, *NEAT* Nuclear Paraspeckle Assembly Transcript 1, *OIP5-AS1* OIP5 Antisense RNA 1, *PEX5L* Peroxisomal Biogenesis Factor 5 Like, *ST7-AS1* ST7 Antisense RNA 1, *TP53COR1* Tumor Protein P53 Pathway Corepressor 1, and *TUG1* Taurine up-regulated gene-1

The differentiation of Ad-MSCs into IPCs was found to be associated with significant elevation in the expression level of MALAT1 by 7.21 and 9.59 folds at step-1 and step-2, respectively, as compared to un-induced control Ad-MSCs as shown in Fig. 4A. Likewise, TUG1 expression levels were significantly upregulated during differentiation by 3.61 and 3.66 folds at step-1 and step-2 induced cells, respectively, as compared to un-induced control Ad-MSCs as shown in Fig. 4B.

TUG1 expression levels were significantly upregulated during differentiation by 3.61 and 3.66 folds at step-1 and step-2 induced cells, respectively, as compared to un-induced control Ad-MSCs as shown in Fig. 4B.

### The interplay between MALAT1 and TUG1 in generated networks by RAIN database

To investigate the potential regulatory landscape of MALAT1, we utilized RNA–protein association and interaction networks (RAIN) (v1.0) to generate an interaction network, revealing associations with other key lncRNAs (such as TUG1, nuclear paraspeckle assembly transcript 1 (NEAT1), hox transcript antisense intergenic RNA (HOTAIR)), transcription factors (such as ATOH7), and metastasis-related proteins (MMP8), suggesting its broad involvement in chromatin remodeling, transcriptional regulation, and cancer-related pathways as shown in Fig. 4C. An interaction network for TUG1 was also generated using RAIN (v1.0) as shown in Fig. 4D which revealed close associations with lncRNAs such as MALAT1, HOTAIR, tumor protein p53 pathway corepressor 1 (TP53COR1), and growth arrest specific 5 (GAS5), suggesting its possible involvement in regulatory pathways linked to epigenetic modulation, cell cycle regulation, and cancer progression. Thus, the generated networks showed some sort of a possible direct interplay between MALAT1 and TUG1, as well as sharing several common partners within the generated RAIN networks such as TP53COR1, HOTAIR, GAS5 which indicates that they are mostly part of the same regulatory lncRNA cluster. All the details of the detected targets within these generated networks can be found in Supplementary Tables S1 and S2.

## Discussion

In the current study, we isolated Ad-MSCs and induced their differentiation towards IPCs. Our results showed that Ad-MSCs indeed fulfilled all MSCs characteristics, and showed good potential to differentiate into IPCs. Additionally, the diferentiation process was found to be associated with significantly upregulated expression levels of the lncRNAs MALAT1 and TUG1. Besides, both MALAT1 and TUG1 were found to be interrelated with each other, and with several common targets within the generated lncRNA-interaction networks for both of them.

In fact, strategies to create surrogate β-cells for cell therapy of DM have ignited significant excitement over the last couple of decades [36, 37]. This has fueled great interest in deriving IPCs from various types of stem cells, including MSCs [31, 38, 39]. Our results regarding the observed feasible isolation, and relatively homogenous population of isolated Ad-MSCs came in perfect agreement with all the previous reports highlighting the important potential of Ad-MSCs for a wide-array of regenerative medicine applications [28, 40–43]. However, despite all the efforts done so far to generate IPCs from stem cells, several issues need to be carefully addressed, including the pleotropic effects induced by individual inducing factors, together with the complexity of the signaling pathways involved, differences between the various types of used stem cells, and the effect of several factors on the differentiation and therapeutic potential of stem cells [27, 44, 45]. Thus, further exploration in the differentiation of stem cells to IPCs, is indeed warranted together with investigating various molecular mediators interrelated with the differentiation process.

In the current study, we induced the differentiation of Ad-MSCs towards IPCs using induction protocol which incorporated β-mercaptoethanol, nicotinamide, and exendin-4 [29]. Notably, β-mercaptoethanol was included during the early differentiation stage because reducing agents and and redox-modulating conditions have been reported to influence stem-cell differentiation and pancreatic endocrine development by regulating intracellular oxidative balance and differentiation-associated signaling pathways [46, 47]. Whereas nicotinamide and exendin-4 were incorporated during later stages because of their established role in promoting endocrine maturation, insulin synthesis, β-cell differentiation, and functional maturation of insulin-producing cells [31, 48, 49]. During the differentiation process, generated IPCs tended to to detach and form clusters, and showed positive DTZ staining. Interestingly, the generated IPCs showed a profound induction for the expression levels of all investigated β-cell related genes in the current study; *MafA*, *NKX6.1*, and *Ins2* as compared to their respective control un-induced Ad-MSCs. Interestingly, Alongside the significant induction of key pancreatic β-cell markers, we observed a marked upregulation of the lncRNAs MALAT1 and TUG1 during the differentiation process compared to uninduced control cells.

MALAT1 has been reported to regulate gene expression through various mechanisms such as modulation of alternative splicing [50], chromatin remodeling, and transcriptional regulation [51]. Additonally, MALAT1 has been reported to be implicated in mediating the differentiation capacity of MSCs towards lineages such as osteoblasts [52–56], or endothelial cells [57, 58] via various mechanisms. Moreover, MALAT1 has recently been reported to regulate induced pluripotent stem cells (iPSCs)-derived β-cell differentiation by targeting miR-15b-5p/Ihh axis [59], and also to regulate the differentiation efficiency of primary mouse hepatocytes towards IPCs [60]. However, its possible interrelation with differentiation of MSCs towards IPCs has not been investigated until the current study.

Interestingly, MALAT1 was reported to be downregulated in lipotoxicity-induced MIN6 β-cells and in that same study exendin-4 ameliorated β-cell dysfunction, at least partially, via MALAT1 upregulation [23]. This comes in perfect agreement with the observed significantly upregulated levels of MALAT1 in differentiated IPCs compared to uninduced Ad-MSCs in the current study, exhibiting even higher levels after being induced with exendin-4 during phase 2 of the induction protocol. Actually, its upregulation during IPCs differentiation in the current study may reflect its involvement in orchestrating the expression of key transcription factors and β-cell-specific genes, including NKX6.1 and MafA, which are essential for β-cell identity and insulin synthesis [61, 62].

Additionally, MALAT1 may contribute to the differentiation process via possibily modulating signaling pathways which have been linked to β-cell development and function and are known to be activated during IPCs differentiation [63], such as the PI3K/Akt [64], Wnt/β-catenin [65], and TGF-β [66] pathways. Moreover, MALAT1 has been recently reported to function as a competing endogenous RNA (ceRNA), sequestering miRNAs that negatively regulate insulin gene expression and β-cell markers [59, 60]. A recent study by *Wang and co-workers* reported that MALAT1 acts as a ceRNA for miR-15b-5p during differentiation of induced iPSCs into β-like cells, and that downregulation or overexpression of MALAT1 affects insulin production via a MALAT1–miR-15b-5p–Ihh regulatory axis [59]. Another study by *Luo and colleagues* reported that MALAT1 acts as a ceRNA for miR-124-3p, thereby altering PI3K signaling and affecting insulin expression and differentiation efficiency of primary mouse hepatocytes into IPCs [60]. Therefore, the increased expression of MALAT1 during IPCs differentiation may support insulin production and secretion via sponging miRNAs which negatively regulate the expression of insulin and/or β-cell factors regulating its transcription, thereby relieving miRNA-mediated repression of target genes critical for IPCs diferentiation. Thus, MALAT1 upregulation may be part of an adaptive transcriptional network enabling generation and maturation of IPCs from MSCs. However, it’s important to point here that although MALAT1 has been reported to be interrelated with insulin transcription [67], and also with modulating key transcription factors like PDX-1 via mediating histone modifications [68], yet the role of MALAT1 in β-cell function and in human β-cells development in general remains far from complete elucidation [69].

As for our second lncRNA of interest; TUG1, we also observed a significant upregulation of its expression levels during Ad-MSCs differentiation towards IPCs. This suggests that TUG1 may play a regulatory role in IPCs lineage commitment and maturation. Like MALAT1, TUG1 has also been reported to function as a ceRNA, sponging miRNAs such as miR-29a, miR-29c, miR-34a, miR-328 or others [70–74]. Some of these miRNAs have been reported to negatively regulate β-cell-related factors such as miR-29a [75]. Apart from acting as a ceRNA, TUG1 has also been reported to modulate apoptosis and insulin secretion in β-cells; its knockdown caused increased apoptosis and decreased insulin secretion [76]. Furthermore, overexpression of TUG1 has been reported to aleviate the detrimental effects of high glucose/high fat on cell viability and apoptosis in intestinal epithelial cells, suggesting a protective role against metabolic/cellular stress [77]. It’s noteworthy here that oxidative stress is reported to be somehow implicated during the differentiation of stem cells [78–80]. Thus, the observed upregulated expression of TUG1 during differentiation of Ad-MSCs towards IPCs in the current study implies its possible protective role required by the cells to maintain viability and overcome stress during the reprogramming process for generation and maturation of IPCs.

Importantly, our induction protocol incorporated nicotinamide and exendin-4 which were previously reported in other studies to activate/modulate several signaling pathways including PI3K/Akt and/or cAMP/PKA [81–83], and TUG1 has also been previously reported to be directly or indirectly interrelated with these pathways [84, 85]. Thus, the observed TUG1 upregulation may reflect its possible involvement in orchestrating these signaling pathways and facilitating efficient Ad-MSCs commitment to the pancreatic lineage. Moreover, During differentiation towards IPCs, TUG1 may enhance mitochondrial biogenesis/function to support metabolic shifts during IPCs generation and maturation [86, 87]. Additionally, TUG1 is known to interact with epigenetic modifiers such as polycomb repressive complex 2 (PRC2) [88], thereby possibly mediating maintenance and establishment of polycomb repression during differentiation [89]. Thus, like MALAT1, TUG1 upregulation may also be part of some sort of an adaptive transcriptional network enabling generation and maturation of IPCs from MSCs. However, its important to highlight that in the current study, the direct interplay between MALAT1 and TUG1 with various signalling pathways was not experimentally validated, and further thorough mechanistic molecular studies are undoubtedly warranted to fully elucidate the hypothesized network enabling generation of IPCs from MSCs.

In fact, the significant increase in MALAT1 and TUG1 expression during differentiation of Ad-MSCs towards IPCs reflect their active roles in orchestrating this differentiation process, and suggests a possible coordinated transcriptional and/or post-transcriptional network supporting lineage commitment and functional maturation toward a β-cell-like phenotype. Accordingly, to further explore the interplay between MALAT1 and TUG1 with each other, and with other lncRNAs, we generated interaction networks, using bioinformatics analyses, for each of them using RAIN database within the STRING platform. Interestingly, our RAIN-generated interaction networks revealed a close functional interplay between MALAT1 and TUG1, as both of them are embedded within a highly interconnected lncRNA regulatory landscape; directly interacting with each other and sharing multiple critical partners including TP53COR1 , HOTAIR , and GAS5 . This suggests that MALAT1 and TUG1 may cooperatively coordinate chromatin remodeling, transcriptional regulation, and miRNA sponging to co-regulate and coordinate pathways involved in cell cycle progression, apoptosis, and differentiation. It’s noteworthy here that these findings align with previous evidence that both lncRNAs are frequently dysregulated in various cancers, where they may act to enhance tumorigenic potential [90]. Furthermore, ingenuity pathway analysis revealed crosstalk between each of MALAT1 and TUG1 with various molecular mediators interrelated with MSCs differentiation, together with several common targets such as enhancer of z este 2 polycomb repressive complex 2 subunit (EZH2), brain-derived neurotrophic factor) (BDNF), and interleukin-6) (IL-6) as shown in Supplementary Fig. S1.

It’s noteworthy here that the current study has a couple of limitations. First, we used a chemical digestion method using collagenase to isolate Ad-MSCs which might adversely affect their viability [29, 91]. Second, its noteworthy here that although CD73 and CD105 are included in the ISCT minimal criteria for human MSCs, rodent MSCs immunophenotyping is to some extent less standardized and may vary between studies; therefore, MSCs characterization in the present work relied on combined phenotypic adherence, multi-lineage differentiation capacity, and immunophenotypic profiling consistent with previous validated reports [17, 27–30, 43]. Third, the differentiation process was conducted in vitro and we did not investigate the expression levels of MALAT1 and TUG1 during the maturation of generated IPCs upon transplantation in vivo. It is important to highlight here that a profound induction of the expression levels of various β-cell markers has been reported upon transplantation of in vitro-generated IPCs from differentiated MSCs in vivo [92]. Thus, future studies to investigate the possible implication of MALAT1 and TUG1 during such maturation in vivo can undoubtedly provide further insights to their role in differentiation of MSCs into IPCs. Third, the current study is also, to some extent, limited by the lack of functional validation of the potential targets interrelated with MALAT1/TUG1 which were identified via in silico bioinformatics analyses in generated networks by RAIN database. However, identifying these potential mediators via bioinformatics analyses opens the door for a wide-array of future mechanistic molecular investigations which are indeed warranted to fully elucidate the roles of MALAT1 and TUG1 as key molecular mediators.

Taken together, these findings indicate that both MALAT1 and TUG1 may serve as potential novel interrelated molecular mediators regulating the differentiation process of MSCs towards IPCs, adding an additional regulatory layer to the molecular mechanisms underlying IPCs generation from stem cells. A notion that undoubtedly warrant several thorough mechanistic studies to further elucidate their precise roles in β-cell lineage specification and maturation, and opens the door to future studies to determine whether modulating MALAT1 and/or TUG1 levels during differentiation can enhance the efficiency of IPCs generation for therapeutic applications in diabetes.

## Conclusion

Collectively, the findings of the current study suggest that both MALAT1 and TUG1 may serve not only as molecular markers but also as important interrelated regulatory nodes enhancing IPCs generation and maturation from MSCs. Their upregulation during differentiation can be interrelated with ceRNA networks, mediating various epigenetic modifications, orchestrating signaling pathways and overcoming cellular stress during the reprogramming process of Ad-MSCs towards IPCs. In fact, MALAT1 and TUG1 upregulation can be part of some sort of an adaptive transcriptional network together with other molecular mediators enabling generation and maturation of IPCs from MSCs. These insights provide a foundation for future studies to dissect the precise molecular mechanisms by which MALAT1 and TUG1 contribute to β-cell lineage specification, with potential implications for improving stem cell-based therapies for diabetes.

## Supplementary Information

Supplementary Material 1.

## Data Availability

All data generated or analyzed during this study are included in this article and its supplementary files.

## Abbreviations

Ad-MSCs: Adipose tissue-derived mesenchymal stem cells
ATOH7: Atonal BHLH transcription factor 7
BDNF: Brain-derived neurotrophic factor
BLACAT1: Bladder cancer-associated transcript 1 (Non-protein coding)
CASC7: Cancer susceptibility candidate 7
CD: Cluster of differentiation
ceRNA: Competing endogenous RNA
DM: Diabetes mellitus
DMEM: Dulbecco’s modified Eagle medium
DMSO: Dimethyl Sulfoxide
DTZ: Dithizone
ESCs: Embryonic stem cells
EZH2: Enhancer of zeste 2 polycomb repressive complex 2 subunit
FBS: Fetal bovine serum
FITC: Fluroisothiocyanate
GAS5: Growth arrest specific 5
HCN2: Hyperpolarization activated cyclic nucleotide gated potassium channel 2
HCN3: Hyperpolarization activated cyclic nucleotide gated potassium channel 3
HCN4: Hyperpolarization activated cyclic nucleotide gated potassium channel 4
HG: High glucose
HOTAIR: Hox transcript antisense intergenic RNA
IL-6: Interleukin-6
Ins2: Insulin
IPCs: Insulin-producing cells
iPSCs: Induced pluripotent Stem Cells
ISCT: International society for cellular therapy
LINC00229: Long intergenic non-protein coding RNA 229
LINC00493: Long intergenic non-protein coding RNA 493
LncRNAs: Long non-coding RNAs
MafA: V-maf avian musculoaponeurotic fibrosarcoma oncogene homolog A
MALAT1: Metastasis-associated lung adenocarcinoma transcript-1
MMP8: Matrix metallopeptidase 8
MSCs: Mesenchymal stem cells
NEAT: Nuclear paraspeckle assembly transcript 1
NKX6.1: Homeobox protein NKX6.1
OIP5-AS1: OIP5 antisense RNA 1
PBS: Phosphate buffered saline
PDX-1: Pancreatic and duodenal homeobox 1
PE: Phycoerythrin
PEX5L: Peroxisomal biogenesis factor 5 like
RAIN: RNA–protein association and interaction networks
RT-qPCR: Reverse transcriptase quantitative polymerase chain reaction
ST7-AS1: ST7 antisense RNA 1
SVF: Stromal vascular fraction
TP53COR1: Tumor protein P53 pathway corepressor-1
TUG1: Taurine upregulated gene-1
