# Supplementary material for: Emerging roles of the long non-coding RNAs MALAT1 and TUG1 during differentiation of adipose tissue-derived mesenchymal stem cells towards insulin-producing cells
Source: Stem Cell Res Ther. 2026 Jul 1;17:233. doi: 10.1186/s13287-026-05125-y (PMC13325589; doi:10.1186/s13287-026-05125-y)
Supplement: Supplementary file 1 — Supplementary Material 1. [file 13287_2026_5125_MOESM1_ESM.pdf]

Supplementary materials

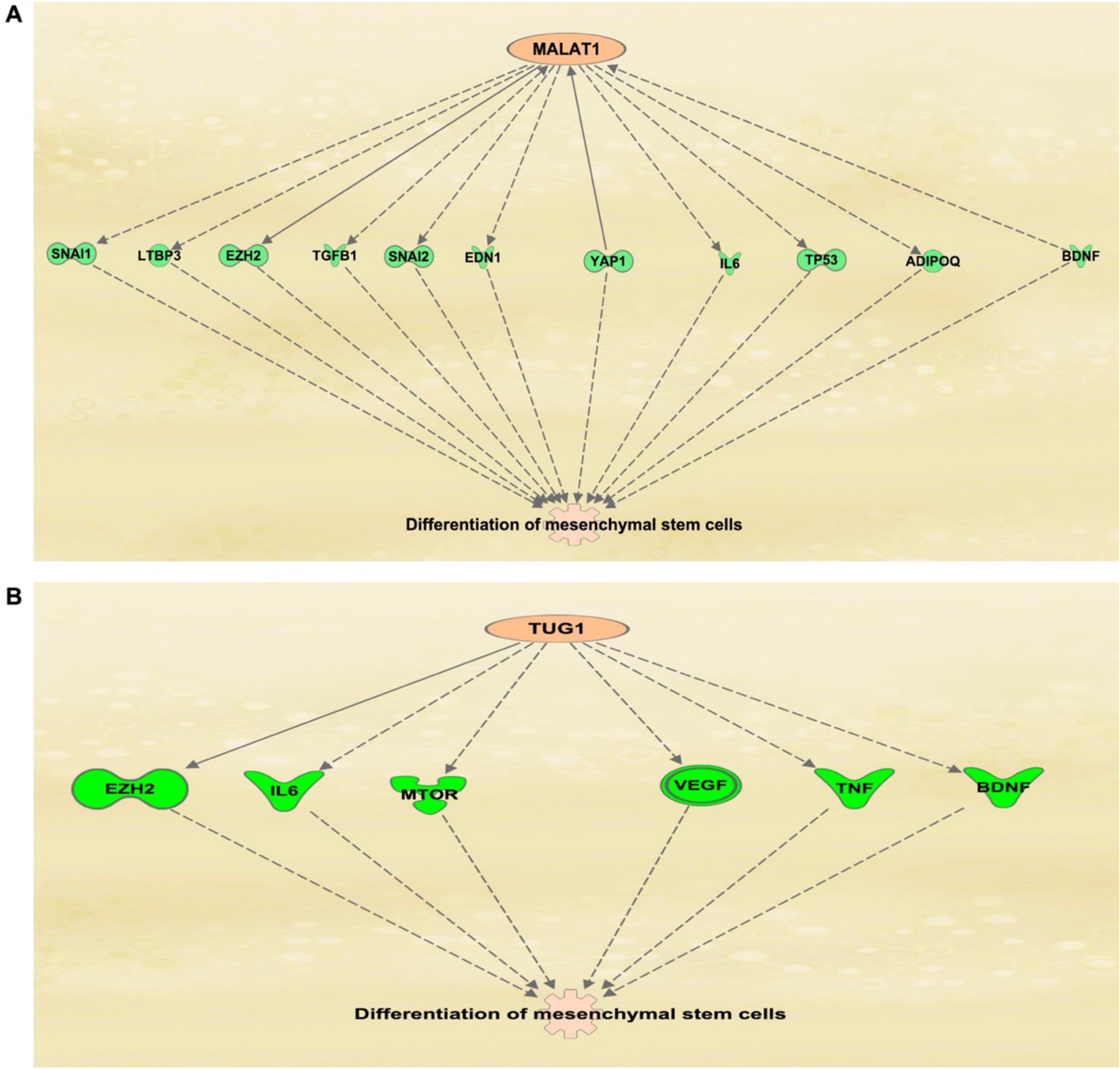

Supplementary Figure S1: Ingenuity pathway analysis (IPA) reveals possible crosstalk between the lncRNAs MALAT1 and TUG1 with various molecular mediators interrelated with MSCs differentiation. A solid line/arrow represent a direct interaction, while a dotted arrow means there is an indirect interaction(s). The figure was generated using a trial version of Ingenuity Pathway Analysis Software (IPA, Qiagen, The Netherlands). *ADIPOQ, Adiponectin Precursor; BDNF, Brain Derived Neurotrophic Factor; EDN1, Endothelin 1; EZH2, Enhancer Of Zeste 2 Polycomb Repressive Complex 2 Subunit; IL-6, Interleukin-6; LTBP3, Latent Transforming Growth Factor Beta Binding Protein 3; MTOR, Mammalian Target Of Rapamycin; SNAI1, Snail Family Transcriptional Repressor 1; SNAI2, Snail Family Transcriptional Repressor 2; TGF-B1, Transforming Growth Factor Beta 1; TNF, Tumor Necrosis Factor; TP53, Tumor Protein P53; VEGF, Vascular Endothelial Growth Factor; and YAP1, Yes1 Associated Transcriptional Regulator.*

Supplementary Table S1 - MALAT1 RAIN interactions

gene_id1	gene_id2	gene_name1	gene_name2	taxid1	taxid2	combined_score
**9606.ENSP00000261917**	9606.ENSP00000251287	HCN4	HCN2	9606	9606	0.956
**MALAT1**	9606.ENSP00000357342	MALAT1	HCN3	9606	9606	0.76
**9606.ENSP00000236826**	MALAT1	MMP8	MALAT1	9606	9606	0.76
**9606.ENSP00000261917**	MALAT1	HCN4	MALAT1	9606	9606	0.76
**NEAT1**	MALAT1	NEAT1	MALAT1	9606	9606	0.759
**MALAT1**	HOTAIR	MALAT1	HOTAIR	9606	9606	0.758
**TUG1**	TP53COR1	TUG1	TP53COR1	9606	9606	0.756
**TUG1**	MALAT1	TUG1	MALAT1	9606	9606	0.754
**TUG1**	HOTAIR	TUG1	HOTAIR	9606	9606	0.753
**TP53COR1**	HOTAIR	TP53COR1	HOTAIR	9606	9606	0.752
**HOTAIR**	9606.ENSP00000362777	HOTAIR	ATOH7	9606	9606	0.752
**9606.ENSP00000251287**	MALAT1	HCN2	MALAT1	9606	9606	0.752
**TP53COR1**	MALAT1	TP53COR1	MALAT1	9606	9606	0.751
**MALAT1**	9606.ENSP00000362777	MALAT1	ATOH7	9606	9606	0.751
**9606.ENSP00000419975**	MALAT1	PEX5L	MALAT1	9606	9606	0.75
**NEAT1**	TUG1	NEAT1	TUG1	9606	9606	0.742
**NEAT1**	9606.ENSP00000362777	NEAT1	ATOH7	9606	9606	0.733
**TP53COR1**	9606.ENSP00000362777	TP53COR1	ATOH7	9606	9606	0.73
**NEAT1**	TP53COR1	NEAT1	TP53COR1	9606	9606	0.668
**9606.ENSP00000419975**	9606.ENSP00000261917	PEX5L	HCN4	9606	9606	0.653
**TUG1**	9606.ENSP00000362777	TUG1	ATOH7	9606	9606	0.629
**9606.ENSP00000419975**	9606.ENSP00000357342	PEX5L	HCN3	9606	9606	0.55
**NEAT1**	HOTAIR	NEAT1	HOTAIR	9606	9606	0.345
**9606.ENSP00000357342**	9606.ENSP00000261917	HCN3	HCN4	9606	9606	0.34
**9606.ENSP00000419975**	9606.ENSP00000251287	PEX5L	HCN2	9606	9606	0.265
**9606.ENSP00000362777**	9606.ENSP00000236826	ATOH7	MMP8	9606	9606	0.17

Supplementary Table S2 - TUG1 RAIN interactions

gene_id1	gene_id2	gene_name1	gene_name2	taxid1	taxid2	combined_score
**CASC7**	OIP5-AS1	CASC7	OIP5-AS1	9606	9606	0.76
**MALAT1**	HOTAIR	MALAT1	HOTAIR	9606	9606	0.758
**TUG1**	LINC00229	TUG1	LINC00229	9606	9606	0.756
**TP53COR1**	TUG1	TP53COR1	TUG1	9606	9606	0.756
**9606.ENSP00000466433**	TUG1	LINC00493	TUG1	9606	9606	0.756
**TP53COR1**	GAS5	TP53COR1	GAS5	9606	9606	0.754
**MALAT1**	TUG1	MALAT1	TUG1	9606	9606	0.754
**CASC7**	TUG1	CASC7	TUG1	9606	9606	0.754
**TUG1**	HOTAIR	TUG1	HOTAIR	9606	9606	0.753
**TUG1**	GAS5	TUG1	GAS5	9606	9606	0.752
**TP53COR1**	HOTAIR	TP53COR1	HOTAIR	9606	9606	0.752
**TUG1**	BLACAT1	TUG1	BLACAT1	9606	9606	0.752
**TP53COR1**	MALAT1	TP53COR1	MALAT1	9606	9606	0.751
**HOTAIR**	GAS5	HOTAIR	GAS5	9606	9606	0.75
**OIP5-AS1**	TUG1	OIP5-AS1	TUG1	9606	9606	0.75
**ST7-AS1**	TUG1	ST7-AS1	TUG1	9606	9606	0.75
**BLACAT1**	GAS5	BLACAT1	GAS5	9606	9606	0.738
**MALAT1**	GAS5	MALAT1	GAS5	9606	9606	0.735
**ST7-AS1**	GAS5	ST7-AS1	GAS5	9606	9606	0.731
**CASC7**	HOTAIR	CASC7	HOTAIR	9606	9606	0.719
**MALAT1**	9606.ENSP00000466433	MALAT1	LINC00493	9606	9606	0.697
**OIP5-AS1**	HOTAIR	OIP5-AS1	HOTAIR	9606	9606	0.697
**CASC7**	MALAT1	CASC7	MALAT1	9606	9606	0.664
**MALAT1**	BLACAT1	MALAT1	BLACAT1	9606	9606	0.648
**MALAT1**	OIP5-AS1	MALAT1	OIP5-AS1	9606	9606	0.618
**ST7-AS1**	MALAT1	ST7-AS1	MALAT1	9606	9606	0.618
